# Quantitative Evaluation of Dielectric Breakdown of Silicon Micro- and Nanofluidic Devices for Electrophoretic Transport of a Single DNA Molecule

**DOI:** 10.3390/mi9040180

**Published:** 2018-04-13

**Authors:** Mamiko Sano, Noritada Kaji, Qiong Wu, Toyohiro Naito, Takao Yasui, Masateru Taniguchi, Tomoji Kawai, Yoshinobu Baba

**Affiliations:** 1 Department of Biomolecular Engineering, Graduate School of Engineering, Nagoya University, Furo-cho, Chikusa-ku, Nagoya 464-8603, Japan; mamiko.sano@toppan.co.jp (M.S.); qiongqusotsugyoushitayo@yahoo.co.jp (Q.W.); yasui@chembio.nagoya-u.ac.jp (T.Y.); babaymtt@chembio.nagoya-u.ac.jp (Y.B.); 2 Department of Applied Chemistry, Graduate School of Engineering, Kyushu University, Moto-oka 744, Nishi-ku, Fukuoka 819-0395, Japan; 3 Japan Science and Technology Agency, PRESTO, 4-1-8 Honcho, Kawaguchi, Saitama 332-0012, Japan; 4 Graduate School of Engineering, Kyoto University, Katsura, Nishikyo-ku, Kyoto 615-8510, Japan; naito@anchem.mc.kyoto-u.ac.jp; 5 ImPACT Research Center for Advanced Nanobiodevices, Nagoya University, Furo-cho, Chikusa-ku, Nagoya 464-8603, Japan; 6 Institute of Scientific and Industrial Research, Osaka University, 8-1 Mihogaoka-cho, Ibaraki, Osaka 567-0047, Japan; taniguti@sanken.osaka-u.ac.jp (M.T.); kawai@sanken.osaka-u.ac.jp (T.K.); 7 Health Research Institute, National Institute of Advanced Industrial Science and Technology, Takamatsu 761-0395, Japan

**Keywords:** dielectric breakdown, insulation, silicon devices, DNA, electrophoresis

## Abstract

In the present study, we quantitatively evaluated dielectric breakdown in silicon-based micro- and nanofluidic devices under practical electrophoretic conditions by changing the thickness of the insulating layer. At higher buffer concentration, a silicon nanofluidic device with a 100 nm or 250 nm silicon dioxide layer tolerated dielectric breakdown up to ca. 10 V/cm, thereby allowing successful electrophoretic migration of a single DNA molecule through a nanochannel. The observed DNA migration behavior suggested that parameters, such as thickness of the insulating layer, tolerance of dielectric breakdown, and bonding status of silicon and glass substrate, should be optimized to achieve successful electrophoretic transport of a DNA molecule through a nanopore for nanopore-based DNA sequencing applications.

## 1. Introduction

Micro- and nanofabrication of silicon semiconductors is well established and is generally easier than fabrication of glass or quartz. However, application of an electric potential on a silicon microchannel can lead to the formation of bubbles or loss of current in the channel during electrophoresis [1]. This phenomenon is caused by electric breakdown of the silicon wafer when subjected to a strong electric field, and represents the most unfavorable feature of silicon microfluidic devices for analytical and bioanalytical chemistry applications. Hence, extensive research efforts in the materials industry have been focused on the development of substrates, such as the development of non-conductive layers using silicon and polymers. To eliminate the intrinsic properties of Si as a semiconductor, Si near the surface is often converted to SiO_2_ through methods such as dry and wet thermal oxidation [2], low-pressure chemical vapor deposition (CVD) [3], and plasma-enhanced CVD [4], prior to bonding with a cover substrate. In ambient air, silicon dioxide growth takes place on silicon wafers to about 2 nm thick, however, thermal oxide diffusion penetrating into the silicon substrates creates a thicker SiO_2_ layer up to about 100 nm at 1100 °C, termed dry thermal oxidation. In wet thermal oxidation, the SiO_2_ layer grows up to about 2.5 μm thick, when the temperature is raised to 800 °C and the silicon wafer is exposed to pure steam. Both of the methods are a “non-linear” growth process, because it becomes more difficult for oxygen to penetrate the previously formed SiO_2_ layer. By contrast, CVD is a linear growth process where a precursor gas deposits a thin film onto a silicon wafer rather than growth of a layer. Therefore, it can produce wide thickness range of SiO_2_ layer. Although all of the resulting surface effectively acts as “glass,” the oxide layer itself can break down when subjected to a very high electric potential. As a rule of thumb, a few hundred nanometer-scale oxidized silicon devices are normally operated at 100 V/cm or lower [5,6]. Therefore, improving the electric tolerance of the silicon dioxide layer on the silicon wafer and precise control of electrokinetic properties on the surface, such as electro-osmotic flow, are critical for electrophoretic experiments in chemical and biochemical analysis. For electrophoresis applications, the surfaces of these devices can be further modified via the Hjerten method [7] to suppress electro-osmotic flow and non-specific sample adsorption to the walls. A high-ionic strength buffer, such as 5× TBE (pH = 8.3; 0.445 M Tris, 0.445 M boric acid, 0.01 M EDTA) also appears to suppress electro-osmotic flow in the absence of any additional surface treatment [8,9]. The use of polymer substrates is another alternative for insulating microfluidic devices, and is particularly suitable for electrophoretic experiments because they enable high voltage operation and are easily fabricated [10].

Our goal was to integrate the micro- and nanofluidic device with a nanopore on a single chip and perform DNA or RNA sequencing [11]. Therefore, the device should be fabricated on a silicon substrate containing nanometer-wide slits to prepare stretched DNA or RNA molecules while taking their lengths into account to allow smooth movement into the nanopore without clogging the pore entrance. In the present study, we demonstrated that electric tolerance of micro- and nanofluidic devices can be significantly increased via deposition of a thick SiO_2_ layer on Si substrate via plasma CVD combined with the use of a concentrated buffer under practical electrophoretic conditions. Considering coexist of different sizes of micro- and nanoscale channels in the integrated device, which might be fabricated by two or more lithographic techniques, three different thickness of SiO_2_ layer were investigated, natural oxide, 100 nm, and 250 nm. Silicon nitride membrane is the most widely used nanopore in DNA sequencers, and has an electric tolerance of ~10 MV/cm, which is an order of magnitude higher than that of silicon dioxide [12]. Although sealing micro- and nanofluidic devices is essential for the construction of three-dimensionally confined channel structures, the silicon nitride layer is not suitable for the subsequent bonding process with cover glass. Thus, the greatest challenge in producing this integrated device is to maintain the electric tolerance, even after bonding of the silicon substrate and glass cover. Post-generation of the silicon dioxide layer inside a silicon micro- or nanochannel cannot be performed using the currently available techniques. Hence, a good bonding method that can preserve the SiO_2_ layer while preventing the formation of defects is crucial during device fabrication. The anodic bonding technique used in this study is an electrical and thermal process that is widely used for sealing silicon substrates with glass [13]. Considering the practical applications and suitability for mass production of the electrophoretic device, TBE buffer, which is the most widely used buffer in electrophoresis experiments, was used to fill the microchannel connected to the nanochannels. In fact, there is a still gap between the solution used in electrophoretic device and the nanopore-based DNA sequencer. While from 10 mM to 1 M KCl solution is generally used to produce ionic current in the nanopore-based DNA sequencer, 0.5× to 5× TBE buffer (pH = 8.3; 44.5–445 mM Tris, 44.5–445 mM boric acid, 1–10 mM EDTA) is a golden standard for electrophoresis. In this work, we used TBE buffer as a model case because the ionic strength of the above two solutions do not have significant difference, 0.01~1. We evaluated the compatibility of our integrated electrophoretic device for future electrophoresis and sequencing applications.

## 2. Materials and Methods

### 2.1. Device Fabrication

**Figure 1:**
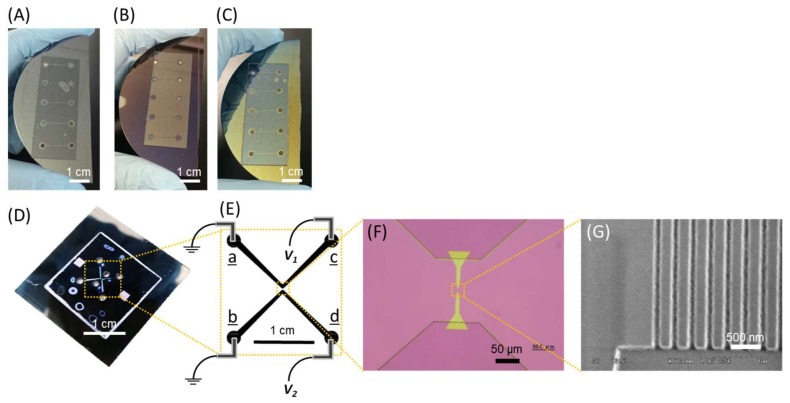
Microfluidic devices for dielectric breakdown experiments (**A**–**C**) and a nanofluidic device for single DNA molecule observation (**D**–**G**). (**A**–**C**) Image of microfluidic devices with (**A**) natural oxide (NO) layer, (**B**) 100 nm thick, and (**C**) 250 nm thick silicon dioxide layer on the surface of silicon substrate formed via CVD. The dimension of all the microchannels were; 200 μm in width, 2 μm in depth, and 1 cm in length. (**D**) Image of the nanofluidic devices. Micro- and nanochannels were fabricated on a silicon substrate and covered with glass via anodic bonding. (**E**) Schematic illustration of the micro- and nanochannels and the electrodes setup. (**F**) Optical microscope image of the nanochannels bridging two wide microchannels. The highlighted region by yellow indicates the area fabricated by EB lithography. (**G**) Scanning electron microscope image of the nanochannels. Both the width and the depth of the nanochannels are 100 nm and the length is 10 μm.

To study the dielectric breakdown of the silicon microchip, devices were fabricated using standard lithographic methods. Phosphorus-dope n-type silicon wafers (diameter: 76 ± 0.5 mm, thickness: 380 ± 25 μm, resistivity: 1–10 Ω∙cm, direction: <100> ± 1°) were purchased from Advantec Co., Ltd. (Tokyo, Japan). The silicon wafer was coated with OAP (Tokyo Ohka Kogyo Co., Ltd., Kawasaki, Japan) as a primer to improve adhesion between a photoresist and a silicon wafer, coated with positive photoresist OFPR-8600LB (Tokyo Ohka Kogyo Co., Ltd., Kawasaki, Japan) using a spin coater, and pre-baked at 95 °C for 5 min. The wafer was exposed to UV light through a photomask and then developed for 1 min in 2.38% NMD-3 solution (Tokyo Ohka Kogyo Co., Ltd., Kawasaki, Japan). The device was heated on a hot plate at 120 °C for 3 min, and the resulting photoresist pattern was delineated via inductively coupled plasma etching (RIE-800iPB, Samco Inc., Kyoto, Japan). The remaining resist was removed in Stripper 104 solution (Tokyo Ohka Kogyo Co., Ltd., Kawasaki, Japan). The inlet and outlet reservoirs (2 mm each) were drilled using an ultrasonic drill (Drillmaster model SOM-121, Shinodatool Co., Ltd., Okinawa, Japan). The following silicon dioxide layers with varying thicknesses were deposited using an electron cyclotron resonance (ECR) plasma CVD system (AFTEX-3420, JSW AFTY Corporation, Tokyo, Japan): natural oxide, 100 nm thick, and 250 nm thick layers. Each silicon wafer was then washed with piranha solution and standard clean 1 (SC1) solution before bonding with a glass cover. The custom-made anodic bonding instrument was used to bond the silicon wafer and Hoya SD2 glass (Hoya Candeo Optronics Corp., Saitama, Japan). The silicon wafer and SD2 glass were contacted with graphite electrodes (ISO-63, Toyo Tanso Co., Ltd., Tokyo, Japan) and heated to 400 °C on a hot plate, after which a voltage of 1.5 kV was applied for 30 min. After anodic bonding, the wafer was cooled to room temperature and cut using a wafer dicer, as shown in Figure 1A–C. 

For direct observation of a single DNA molecule in a nanochannel, the nanofluidic devices were fabricated via standard EB lithography and photolithography (Figure S1). Nanochannels with a height of 100 nm and width of 100 nm were used (Figure 1D–G).

### 2.2. Current Measurements

For measurement of current–voltage curves, Pt electrodes were inserted into the reservoirs filled with buffer, and electric fields were applied in a stepwise manner using an external high voltage sequencer (HVS448 1500V, LabSmith, Inc., Livermore, CA, USA). Various concentrations of TBE buffer ranging from 1× to 5× TBE (44.5 mM Tris, 44.5 mM Borate, and 1 mM EDTA) were prepared and used for the current measurements. 

Conductivity of the TBE buffer was measured at 25 °C using a conductivity meter (DS-52, HORIBA, Ltd., Kyoto, Japan) several times daily (intraday, *n* = 4) over several days (interday, *n* = 3) as shown in Figure S2.

### 2.3. DNA Observation

Bacteriophage T4 GT7 DNA (165.6 kbp) was purchased from Nippon Gene (Tokyo, Japan). T4 GT7 DNA was stained by YOYO^TM^-1 (Thermo Fisher Scientific K.K., Yokohama, Japan) at a dye-to-base ratio of one to five for direct observation. Electrophoretic migration behavior of the DNA molecules in the nanochannels was observed under an inverted fluorescent microscope (Eclipse Ti, Nikon Corporation, Tokyo, Japan) and recorded on a DV tape (Sony DV 180 ME Digital Video Cassette, Sony Corp., Tokyo, Japan).

## 3. Results and Discussion

**Figure 2:**
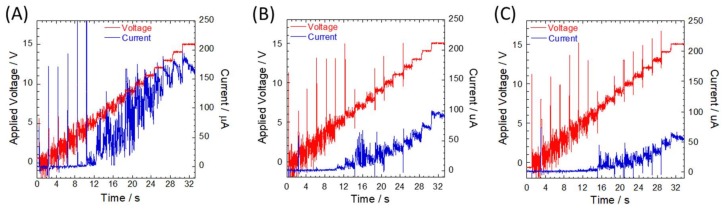
Representative time courses of the applied voltage (red line) and the channel currents (blue line) on the silicon microchannels with the following varying oxide layer thicknesses: (**A**) NO, (**B**) 100 nm, and (**C**) 250 nm. In all experiments, channels were filled with 1× TBE, and applied electric fields were raised by 1 V every 2 s from 0 to 15 V. Channel currents were monitored using an ampere meter embedded in the high voltage sequencer. Current measurements were performed in two separate measurements using a single device, namely, from 0 to 7 V and from 8 to 15 V, due to limitations in the programmable voltage sequencer. Therefore, presented values are combined data from the two sets of measurements.

**Figure 3:**
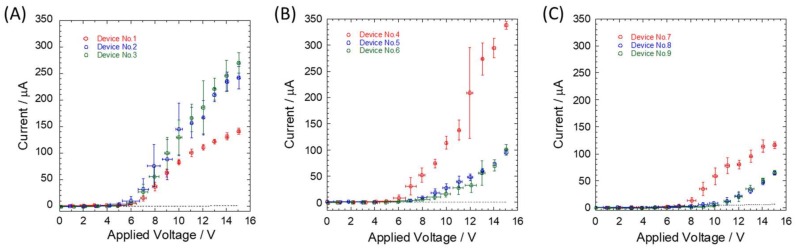
Monitored time-average currents were plotted as a function of the applied voltage. (**A**) NO, (**B**) 100 nm, and (**C**) 250 nm thick oxide layers on the silicon microchannels were filled with 5× TBE. Currents were measured in nine distinct devices, which are depicted in red, blue, and green circles, respectively. Dotted lines show theoretical currents under the assumption that no dielectric breakdown occurs and an electrical current pass through a microchannel.

For quantitative evaluation of the effectiveness of the silicon dioxide layer as an insulation layer, the applied voltage was raised by 1 V every 2 s from 0 to 15 V in a stepwise manner (Figure 2). Although the current was maintained at a constant value under a certain electric field, the current sharply increased over a critical value and showed fluctuation noise. For example, using 5× TBE buffer, voltage conditions of 5, 6, and 7 V correspond to electric fields of 5, 6, and 7 V/cm, respectively, for natural oxide layer, 100 nm oxide layer, and 250 nm oxide layer, respectively. The spike noises observed during voltage switching can be attributed to intrinsic issues in the hardware. Values for dielectric breakdown were determined at the point when the current jumped beyond the theoretically predicted line, as shown in Figure 3 and Figure S3. Compared to a previously reported value of 80 V for a 0.42 cm × 2.0 cm channel filled with phosphate buffer (48 mM Na_2_HPO_4_ and 21 mM KH_2_PO_4_, pH 7) [1], our obtained values were too low. One possible reason is that the surface of the silicon device used in the previous study was prepared using a 207 nm layer of dry thermal oxide, which produces an Si–SiO_2_ interface with excellent electrical properties, whereas the silicon dioxide layer on the surface of our device was deposited via ECR plasma CVD. Ramsey et al. also measured dielectric strength of microchip devices using a microchannel filled with 10 mM sodium tetraborate buffer [14]. Dielectric breakdown was observed at the applied voltage of 15 kV, which corresponds to 1100 kV/cm across a 150 μm sealed gap between the two microchannels by curing at 500 °C after surface hydrolysis. Good mechanical strength of around 6.5 J/m^2^ surface energy showed positive evidence of chemical bonding between the silicon and glass surfaces for microfluidic experiments. The mechanical strength of glass–SiO^2^ bonding was not measured here, but the anodic bonding process employed in this study could have affected integrity of the surface chemical structure and caused molecular defects.

Although this kind of molecular defect in the SiO_2_ layer results in reduced performance of the resulting nanofluidic device, this principle was successfully applied to fabrication of nanopores with diameters as low as 2 nm [15]. A transmembrane potential was applied through the thin SiNx membrane supported by a Si rim, resulting in the accumulation of charge traps inside the SiNx, which in turn induced bond breakage or energetic charge carriers that induced the formation of a highly localized conductive path, followed by a discrete dielectric breakdown event. Smaller nanopores with diameters ranging from 1 to 2 nm generated via dielectric breakdown were further developed by Yanagi et al. in a process called “multilevel pulse-voltage injection” (MPVI) [16]. In this nanopore fabrication process, leakage current was gradually increased, followed by the capacitive spike at nanopore creation under constant transmembrane potential, resulting in increased nanopore size. Considering that the phosphorus-doped n-type silicon wafer used in our experiment exhibits similar semiconducting properties with the n-type SiNx membrane used in nanopore fabrication, structural defects formed in the phosphorus-doped n-type silicon wafer after dielectric breakdown could increase over time. However, an increment in leakage current corresponding to the size of defects after the dielectric breakdown showed an opposite trend to the results of our SiO_2_ experiment. During stepwise increments of the applied voltage, the currents gradually decreased during the 2 s holding state after dielectric breakdown (Figure 2). Assuming that the phenomenon is based on Ohm’s law, resistance can be gradually increased over time because the applied voltage was constant. One possible explanation is that oxidation of the silicon wafer progressed after dielectric breakdown. When defects were formed in the SiO_2_ layer on the Si wafer, a new electrical circuit was formed through the Si wafer other than a microchannel, which has a considerably lower resistivity than the silicon dioxide layer. In the initial stages, the defects could be “a nanometer hole”, and the electric field is concentrated around the hole. As a result, Joule heating leads to bubble formation in the water and the following subsequent wet oxidation process:

Si + 2H_2_O → SiO_2_ + 2H_2_

Compared with that of dry oxidation, the rate of wet oxidation is known to be drastically accelerated to few hundreds at 800 °C [17]. Although we cannot measure the temperature at the time when defects in SiO_2_ layer are formed, the observed slight increase in the current could be due to the regeneration of silicon dioxide at the site of defect within seconds. 

Although the plots in Figure 3 present typical results of single-step increments over the critical applied voltage, more than two-step increments of the currents were observed in some cases (Figures S3–S6). This could be attributed to manner of defect formation in the silicon dioxide layer or the distance between defects, which established electrical conduction. Given that insulation defects were randomly formed when voltage was applied to the microchannel between the reservoirs, multiple electrical paths were formed at different locations at varying time points on the silicon substrate beyond silicon dioxide layer. This phenomenon could have been responsible for the observed multiple-step increments of the current–voltage (I–V) curves.

**Figure 4:**
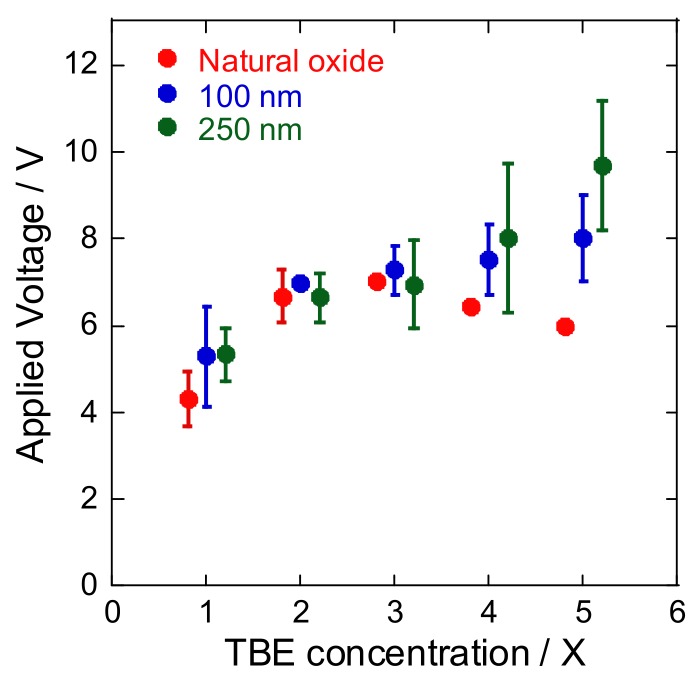
Average voltage applied at the time of dielectric breakdown under different oxide layer thicknesses and buffer concentrations (*n* = 3). At the same concentration of TBE buffer, three plots and error bars depicted in red, blue, and green were intentionally shifted, so as to be easily seen.

The choice of buffer is a critical factor for electrophoresis-based separation techniques. In this study, we used varying concentrations of TBE buffer (Tris–borate–EDTA), which is commonly used in procedures involving nucleic acids. As shown in Figure 4, a more concentrated and thicker dioxide layer had higher critical voltages of dielectric breakdown, except for the microfluidic device fabricated with natural oxide layer. These results are expected, since a more concentrated solution has lower resistance and accommodates larger currents. However, in the case of the natural oxide layer, the critical voltage of dielectric breakdown decreased below 3 V. This tendency reminds us that pH and salt concentrations of a solution markedly influence nanopore fabrication [15]. In nanopore fabrication based on dielectric breakdown, lower pH and higher salt concentrations decreased the fabrication time. Although the total time of voltage application in our measurement was only 30 s, the high TBE buffer concentration could have been sufficient to produce structural defects on the silicon dioxide layer and induce dielectric breakdown.

**Figure 5:**
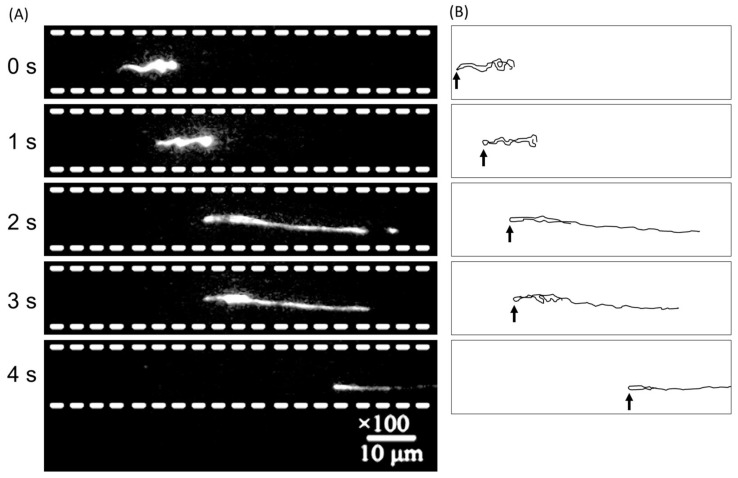
(**A**) Electrophoretic migration behavior of a single T4 DNA molecule in a 100 nm wide and 100 nm deep nanochannel, which is arrayed continuously side-by-side with 400 nm spacing inside dashed white lines. An electric field of around a few V/cm was applied horizontally along the nanochannels. (**B**) Presumed DNA conformation is depicted as a black line in the right for clarity. The folded and twisted parts, indicated by arrows, could be caused by defects in the silicon dioxide layer during anodic bonding. The DNA molecule appears to switch the nanochannel vertically during migration.

Figure 5 shows images of DNA electrophoretic migration in 100 nm wide and 100 nm deep nanochannel coated with 400 nm thick SiO_2_ layer via CVD. The applied voltage was adjusted by incorporating the all the abovementioned factors to optimize the parameters and avoid dielectric breakdown. The molecular behavior of T4 DNA was then observed. The thick silicon dioxide layer (400 nm) and concentrated buffer (5× TBE) successfully prevented dielectric breakdown and facilitated entry of the DNA molecule inside the 100 nm nanochannel. DNA stretching due to polymer confinement was observed from 2 to 4 s. However, at the same time contraction (0 to 1 s), movement of the DNA across a fence to the adjacent nanochannel (1 to 2 s) was observed. This phenomenon could have been caused by surface roughness and impaired anodic bonding between the thick silicon dioxide layer and the SD2 glass. Although correct anodic bonding in nanofluidic devices is generally evaluated by loading fluorescence dyes, such as rhodamine 6G3 and fluorescein [18], the amount of fluorescent dyes released from nanometer-scale defects was too small for direct observation. By contrast, the migration of a DNA molecule labelled with high-density dyes can be easily traced and can help identify the defect during anodic bonding, which is indicated by the presence of a 2 nm hole that allows the movement of a DNA molecule. The result also suggest that a DNA molecule can contribute as a Si defect indicator in nanometer scale. Results showed that the thickness of the silicon dioxide layer is a trade-off between tolerance of dielectric breakdown and perfect silicon-glass bonding. Optimization of the thickness of the silicon dioxide layer in nanofluidic devices is a critical process that depends on the application, and should involve selection of parameters such as sample, buffer, and electric field.

## 4. Conclusions

The insulating layer formed via natural oxidation and plasma chemical vapor deposition (CVD) on silicon devices was quantitatively evaluated by applying an electric field to the microchannel filled with varying concentrations of electrophoretic buffer (1× to 5× TBE) under practical conditions. Although dielectric breakdown was observed at around 10 V/cm in all fabricated devices, a thicker silicon dioxide layer and a more concentrated buffer effectively suppressed dielectric breakdown under high voltage conditions, except for the device fabricated with a natural oxide layer. Our findings helped identify important parameters for future development of Si-based nanopore devices integrated with DNA stretching process as a pretreatment step for a single DNA or RNA sequencing.

## Supplementary Materials

The following are available online at http://www.mdpi.com/2072-666X/9/4/180/s1, Figure S1: Fabrication process of the nanofluidic devices; Figure S2: Conductivities of TBE buffer preparations with varying concentrations at 25 °C (*n* = 12); Figure S3: Monitored time-average currents were plotted as a function of the applied voltage; Figure S4: Monitored time-average currents were plotted as a function of the applied voltage; Figure S5: Monitored time-average currents were plotted as a function of the applied voltage; Figure S6: Monitored time-average currents were plotted as a function of the applied voltage.

## References

[1] Harrison D.J., Glavina P.G., Manz A. (1993). Towards miniaturized electrophoresis and chemical-analysis systems on silicon—An alternative to chemical sensors. Sens. Actuators B Chem..

[2] Xia Q., Morton K.J., Austin R.H., Chou S.Y. (2008). Sub-10 nm self-enclosed self-limited nanofluidic channel arrays. Nano Lett..

[3] Kutchoukov V.G., Laugere F., van der Vlist W., Pakula L., Garini Y., Bossche A. (2004). Fabrication of nanofluidic devices using glass-to-glass anodic bonding. Sens. Actuators A Phys..

[4] Fu J., Mao P., Han J. (2009). Continuous-flow bioseparation using microfabricated anisotropic nanofluidic sieving structures. Nat. Protoc..

[5] Bakajin O., Duke T.A.J., Tegenfeldt J., Chou C.F., Chan S.S., Austin R.H., Cox E.C. (2001). Separation of 100-kilobase DNA molecules in 10 seconds. Anal. Chem..

[6] Fu J., Mao P., Han J. (2005). A nanofilter array chip for fast gel-free biomolecule separation. Appl. Phys. Lett..

[7] Hjerten S., Wu B.L. (1985). Studies of fish zona pellucida by high-performance ion-exchange chromatography on agarose columns and free zone electrophoresis. J. Chromatogr..

[8] Han J., Craighead H.G. (1999). Entropic trapping and sieving of long DNA molecules in a nanofluidic channel. J. Vac. Sci. Technol. A Vac. Surf. Films.

[9] Kaji N., Oki A., Ogawa R., Takamura Y., Nishimoto T., Nakanishi H., Horiike Y., Tokeshi M., Baba Y. (2007). Influences of electroosmotic flows in nanopillar chips on DNA separation: Experimental results and numerical simulations. Isr. J. Chem..

[10] Duffy D.C., McDonald J.C., Schueller O.J.A., Whitesides G.M. (1998). Rapid prototyping of microfluidic systems in poly(dimethylsiloxane). Anal. Chem..

[11] Di Ventra M., Taniguchi M. (2016). Decoding DNA, RNA and peptides with quantum tunnelling. Nat. Nanotechnol..

[12] Xia L.-Q., Lee P.W., Latchford I., Narwankar P.K., Urdahl R., Nickeles A.S., Achutharaman R., Lewis C.B., Nshi Y., Doering R. (2007). Chemical vapor deposition. Handbook of Semiconductor Manugacturing Technology.

[13] Gerlach A., Maas D., Seidel D., Bartuch H., Schundau S., Kaschlik K. (1999). Low-temperature anodic bonding of silicon to silicon wafers by means of intermediate glass layers. Microsyst. Technol..

[14] Wang H.Y., Foote R.S., Jacobson S.C., Schneibel J.H., Ramsey J.M. (1997). Low temperature bonding for microfabrication of chemical analysis devices. Sens. Actuators B Chem..

[15] Kwok H., Briggs K., Tabard-Cossa V. (2014). Nanopore fabrication by controlled dielectric breakdown. PLoS ONE.

[16] Yanagi I., Akahori R., Hatano T., Takeda K.-I. (2014). Fabricating nanopores with diameters of sub-1 nm to 3 nm using multilevel pulse-voltage injection. Sci. Rep..

[17] Gohma H., Hiroyasu M., Kaji N., Katayama S., Sekijima M., Yamashita Y. (2007). Effects of methotrexate and indomethacin on hind paw swelling and serum levels of inflammatory cytokines in collagen-induced arthritic rats. J. Pharmacol. Sci..

[18] Datta A., Gangopadhyay S., Temkin H., Pu Q., Liu S. (2006). Nanofluidic channels by anodic bonding of amorphous silicon to glass to study ion-accumulation and ion-depletion effect. Talanta.

