# Supplementary material for: Quantitative Evaluation of Dielectric Breakdown of Silicon Micro- and Nanofluidic Devices for Electrophoretic Transport of a Single DNA Molecule
Source: Micromachines (Basel). 2018 Apr 13;9(4):180. doi: 10.3390/mi9040180 (PMC6187859; doi:10.3390/mi9040180)
Supplement: Supplementary file 1 [file micromachines-09-00180-s001.pdf]

Quantitative Evaluation of Dielectric Breakdown of Silicon Micro- and Nanofluidic Devices for Electrophoretic Transport of a Single DNA Molecule

Mamiko Sano, Noritada Kaji, Qiong Wu, Toyohiro Naito, Takao Yasui, Masateru Taniguchi, Tomoji Kawai, Yoshinobu Baba

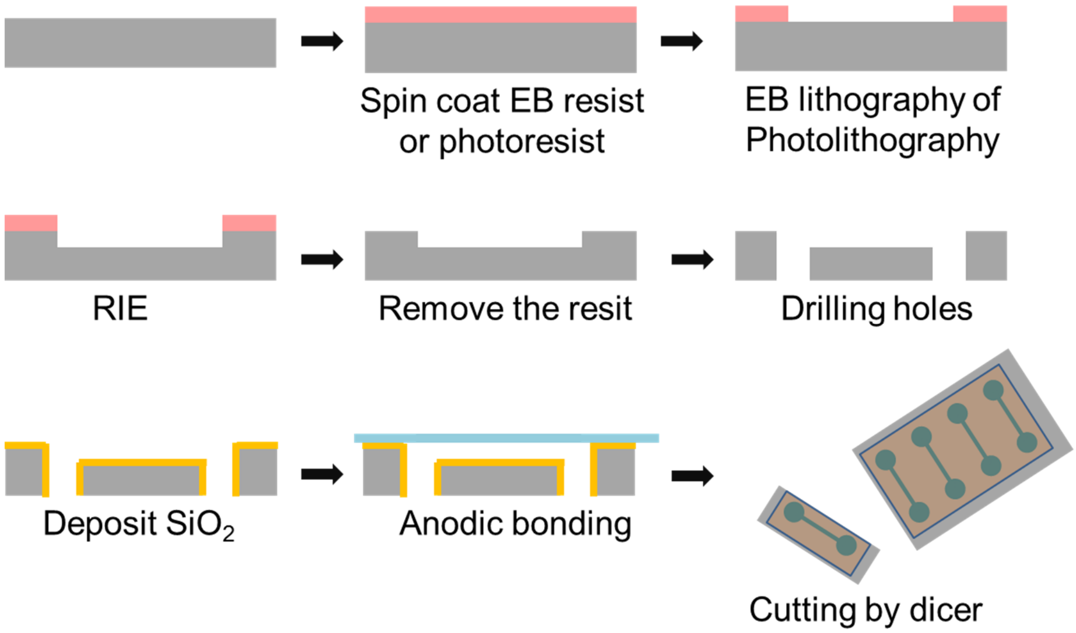

Figure S1. Fabrication process of the nanofluidic devices.

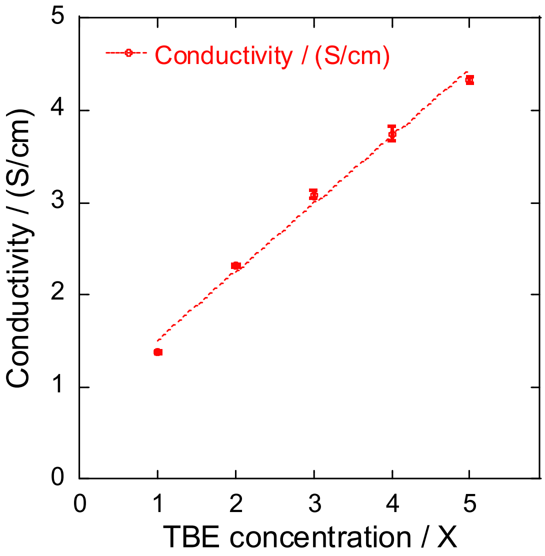

Figure S2. Conductivities of TBE buffer preparations with varying concentrations at 25 °C (n = 12). The dotted line shows the regression line. The correlation coefficient was R = 0.995.

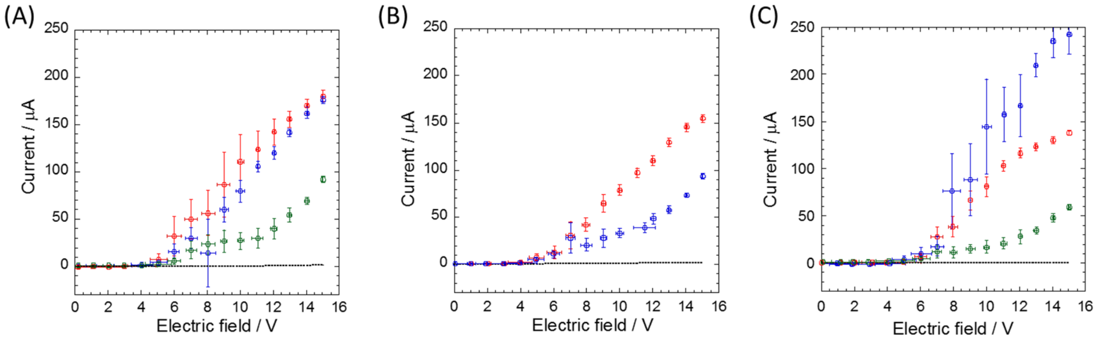

Figure S3. Monitored time-average currents were plotted as a function of the applied voltage. (**a**) NO, (**b**) 100 nm, and (**c**) 250 nm thick oxide layer on the silicon microchannels were filled with 1× TBE. Currents were measured in three different microchannels depicted in red, blue, and red circles, respectively, for each oxide layer condition. Dotted lines show theoretical currents under the assumption that no dielectric breakdown occurs and an electrical current pass through a microchannel.

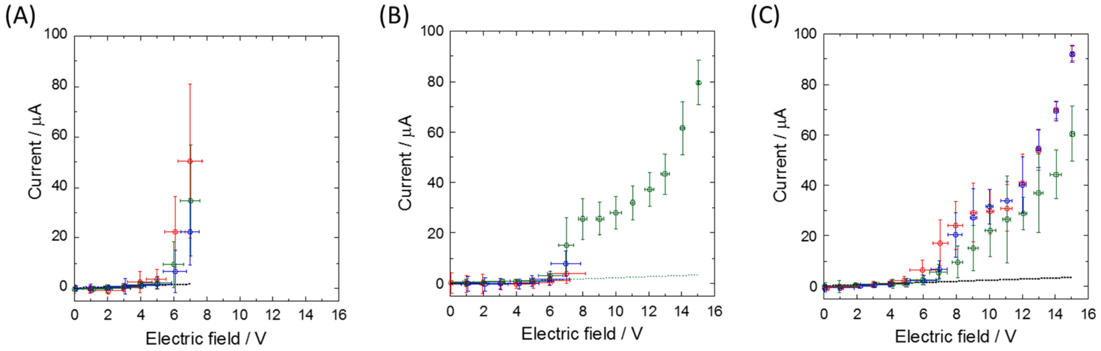

Figure S4. Monitored time-average currents were plotted as a function of the applied voltage. Silicon microchannels with (**a**) NO, (**b**) 100 nm, and (**c**) 250 nm thick oxide layers were filled with 2× TBE. Currents were measured in three different microchannels depicted in red, blue, and red circles, respectively, for each oxide layer condition. Dotted lines show theoretical currents under the assumption that no dielectric breakdown occurs, and an electrical current passes through a microchannel.

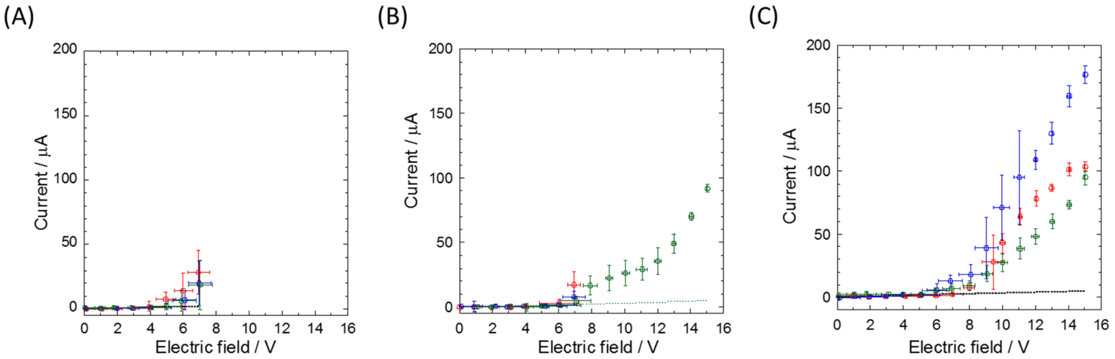

Figure S5. Monitored time-average currents were plotted as a function of the applied voltage. Silicon microchannels with (**a**) NO, (**b**) 100 nm, and (**c**) 250 nm thick oxide layers were filled with 3× TBE. Currents were measured in three different microchannels depicted in red, blue, and red circles, respectively, for each oxide layer condition. Dotted lines show theoretical currents under the assumption that no dielectric breakdown occurs, and an electrical current passes through a microchannel.

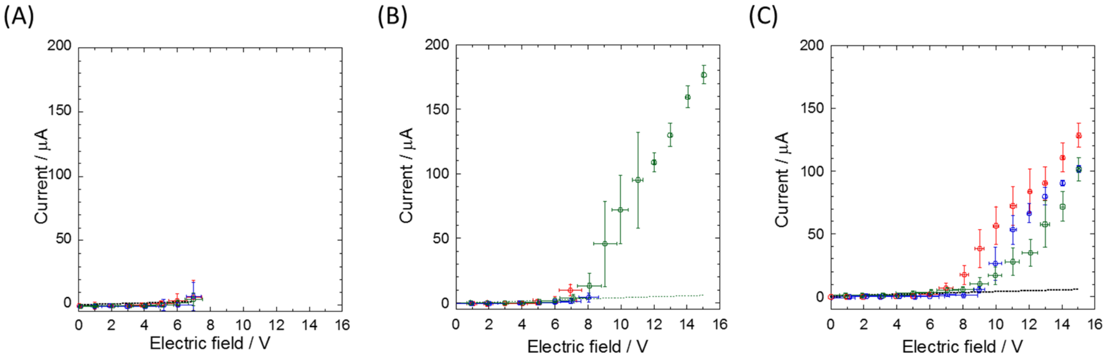

Figure S6. Monitored time-average currents were plotted as a function of the applied voltage. Silicon microchannels with (**a**) NO, (**b**) 100 nm, and (**c**) 250 nm thick oxide layers were filled with 4× TBE. Currents were measured in three different microchannels depicted in red, blue, and red circles, respectively, for each oxide layer condition. Dotted lines show theoretical currents under the assumption that no dielectric breakdown occurs, and an electrical current passes through a microchannel.
